# Size effect on the deformation mechanisms of nanocrystalline platinum thin films

**DOI:** 10.1038/s41598-017-13615-6

**Published:** 2017-10-16

**Authors:** Xinyu Shu, Deli Kong, Yan Lu, Haibo Long, Shiduo Sun, Xuechao Sha, Hao Zhou, Yanhui Chen, Shengcheng Mao, Yinong Liu

**Affiliations:** 1 Beijing Key Lab of Microstructure and Property of Advanced Materials, Beijing University of Technology, Beijing, 100124 China; 2 School of Mechanical and Chemical Engineering, The University of Western Australia, Perth, WA 6009 Australia

## Abstract

This paper reports a study of time-resolved deformation process at the atomic scale of a nanocrystalline Pt thin film captured *in situ* under a transmission electron microscope. The main mechanism of plastic deformation was found to evolve from full dislocation activity-enabled plasticity in large grains (with grain size *d* > 10 nm), to partial dislocation plasticity in smaller grains (with grain size 10 nm < *d* < 6 nm), and grain boundary-mediated plasticity in the matrix with grain sizes *d* < 6 nm. The critical grain size for the transition from full dislocation activity to partial dislocation activity was estimated based on consideration of stacking fault energy. For grain boundary-mediated plasticity, the possible contributions to strain rate of grain creep, grain sliding and grain rotation to plastic deformation were estimated using established models. The contribution of grain creep is found to be negligible, the contribution of grain rotation is effective but limited in magnitude, and grain sliding is suggested to be the dominant deformation mechanism in nanocrystalline Pt thin films. This study provided the direct evidence of these deformation processes at the atomic scale.

## Introduction

Metallic materials may deform plastically in a number of different mechanisms. A general knowledge base has been established of the main plastic deformation mechanisms^1–3^, with the most prevalent being dislocation activities. A well-proven empirical relationship reflecting the interaction between dislocation movement and grain boundaries (GBs) and its effect on strength is the Hall-Petch relationship. However, with the fast advance in recent years in our ability to engineer metallic materials with extremely small grain sizes well beyond what had been possible in the past, it has become clear that this relationship becomes invalid for metals with grain sizes below a certain threshold value^4^. It is understood that at below a certain critical grain size, it is difficult to retain dislocations and to allow normal dislocation activities to operate^2,4^, due to the different mechanics conditions of the matrix at this extremely small scale^5–8^. In this regard, how plastic deformation may occur and how it can be accommodated in nanocrystalline metallic materials need to be understood.

Largely owing to the technical challenges to experimentally study the deformation mechanisms of metals at such small scale, much effort has been made in theoretical analysis. Many molecular dynamics (MD) simulations have been conducted in recent years to investigate the deformation mechanisms in nanocrystalline metallic materials. Some work have suggested that there is a transition from full dislocation activities to grain boundary-mediated deformation with decreasing grain size for metals of high staking fault energies^9–12^. Activation of a grain boundary-mediated deformation mechanism implies that the strength of the metal decreases with the reduction of grain size, i.e., an inverse Hall-Petch correlation. Some MD simulation investigations also suggest that for nanocrystalline metallic materials dislocations emitted from grain boundaries are only partial dislocations^5–8^, and full dislocations^5,9^ are formed by merging the partial dislocations emitted from the same site at grain boundaries. MD simulations^10,13–15^ have also predicted that at *d* < 15 nm only partial dislocations may exist.

To date, there is a severe lacking of direct experimental evidence at the atomic scale in the literature to support any of the hypotheses predicted by MD simulations^5–8^. Experimentally, how nanocrystalline (*d* < ~15 nm) metallic materials may accommodate plastic deformation remains a poorly understood topic, partly because of the technical difficulty to study them with atomic-level resolution^2,3,13,16^. Many previous *in situ* ^17–20^ and *ex situ* ^21–23^ transmission electron microscopy (TEM) experiments have been conducted on thin foil samples with grain sizes of the order of 20 nm or larger. Most of these studies were based on contrast changes observed in bright and dark field TEM images, which are difficult to reveal any possible change-over from dislocation behaviour to grain boundary-mediated plasticity. For nanocrystalline metallic materials with *d* < 15 nm^24,25^, whether there is such a transition of the dominant mechanism of plasticity remains an unanswered question^2,7,25–27^.

In this work, using a home-made device for tensile deformation^27–30^ inside TEM with double-tilt capability to perform *in situ* high-resolution TEM (HRTEM) analysis, we studied the deformation process of a nanocrystalline platinum (Pt) thin films with grain sizes ranging from 20 nm to 3 nm, crossing the regions for both the “Hall-Petch relationship” and the “inverse Hall-Petch relationship”.

However, it is important to understand that the “Hall-Petch effect”, and the follow-on concept of “inverse Hall-Petch effect”, describe the mechanical behavior of bulk materials and rely on the statistical reliability of the microscopic plasticity activities at grain boundaries. Such general conditions found in bulk materials are not duplicated in thin film materials or thin foil samples used for TEM observation, which are often single crystalline (thus lack of grain boundaries) in the thickness direction and which have distinctively different mechanics environments for dislocation activities. In this regard, whereas the “Hall-Petch” and the “inverse Hall-Petch” behaviors may still manifest in thin film materials, which have been experimentally observed^31^, it is reasonable to think that the “Hall-Petch” behavior in thin film materials may not necessarily follow the same mathematical expression as the original “Hall-Petch” equation established for bulk materials. This work, by the nature of TEM observation method, provides a qualitative description of the microscopic mechanisms of the “Hall-Petch” and the “inverse Hall-Petch” behaviors of nanocrystalline Pt thin films.

## Results and Discussions

**Figure 1:**
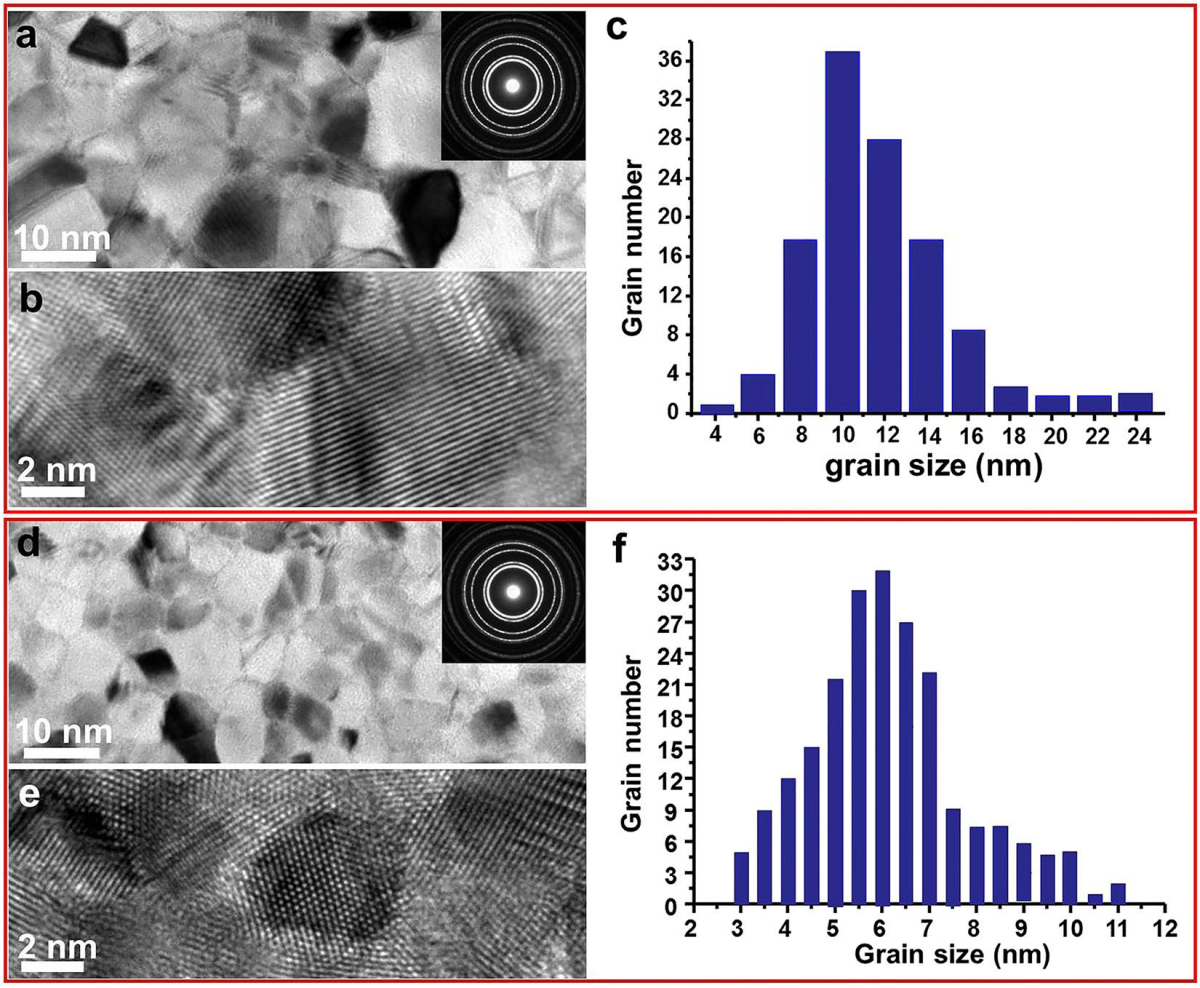
Grain structures of the polycrystalline Pt films. Image (**a**) and (**b**) show the microstructure of film I at low and high magnifications. The inset in (**a**) shows a selected area electron diffraction pattern of the film. Graph (**c**) shows grain size distribution of the film. Images (**d**) and (**e**) show the microstructure of film II at low and high magnifications. The inset in (**d**) shows a selected area electron diffraction pattern of the film. Graph (**f**) shows grain size distribution of the film.

*In situ* tensile deformation was conducted on two nanocrystalline Pt thin films of different grain sizes, one within the “Hall-Petch effect” range (film I) and the other in the “inverse Hall-Petch effect” range (film II). Figure 1a is a TEM micrograph of film I. The film sample contained equiaxial grains with a mean size of 10 nm, as shown in Fig. 1c. Figure 1b shows a HRTEM image of the film. It is seen that the grain boundaries are well crystalline and highly ordered. Figure 1d shows a TEM image of the second Pt thin film with grain sizes in the “inverse Hall-Petch effect” range. The sample contained smaller equiaxial grains of 3 to 11 nm in size, with an average size of ~6 nm. The HRTEM image in Fig. 1e confirms that the GBs are well crystalline and highly ordered.

**Figure 2:**
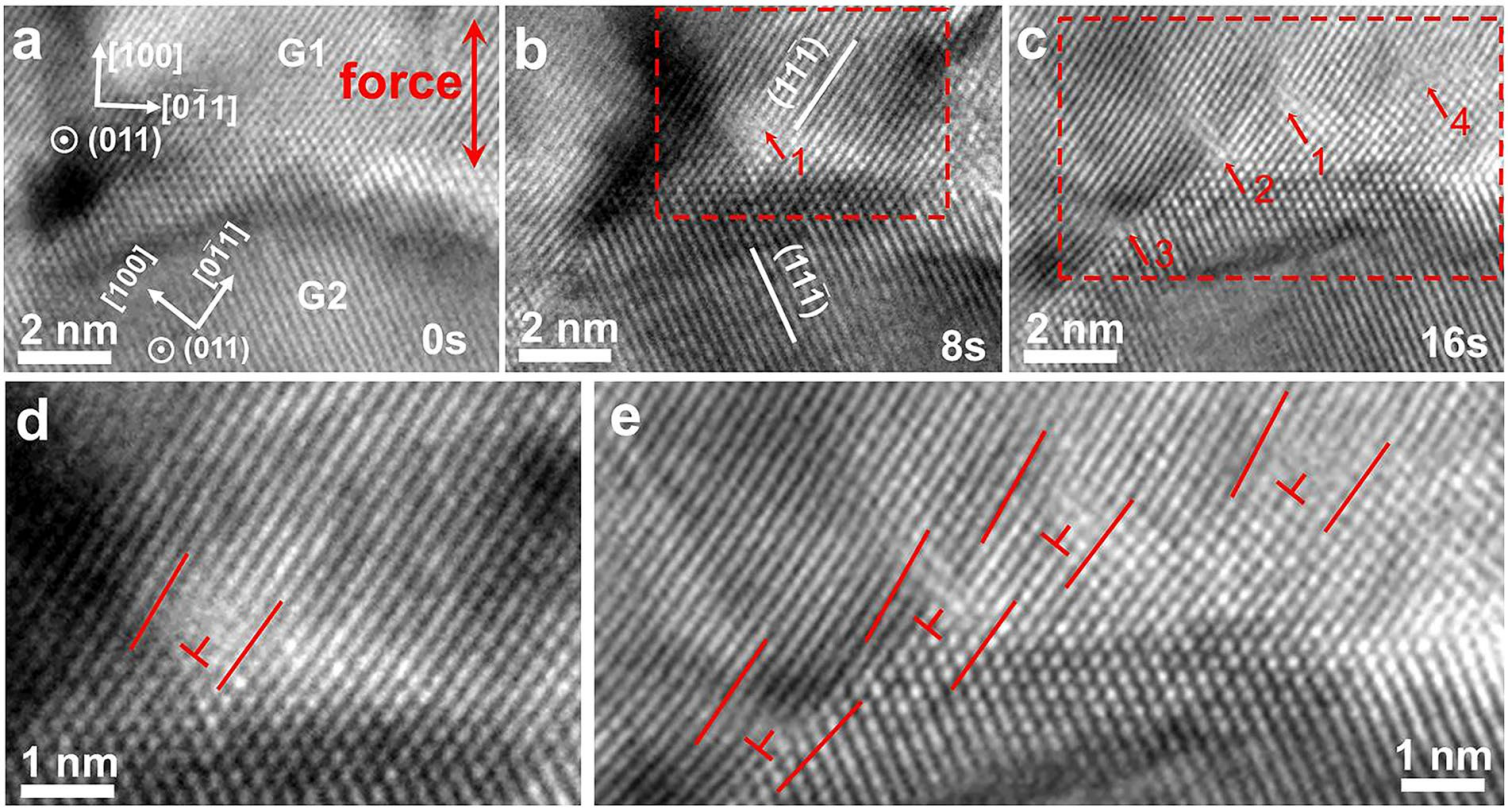
*In situ* atomic-scale observation of dislocation formation in large grains in film I upon straining. The images shown in (**a**), (**b**) and (**c**) were taken at 8 second intervals during straining, showing the progressive nucleation at the grain boundary and movement into the grain interior of full dislocations. (**d**) and (**e**) Show enlarged HRTEM images corresponding to the framed regions in (**b**) and (**c**), respectively.

Figure 2 shows *in situ* observation of film I during tensile deformation. Figure 2a shows an area containing two grains, labeled G_1_ and G_2_. It is evident that both grains display clear lattice fringes, and are dislocation free. It is also evident that the grain boundary between the two, denoted GB_1–2_, is also highly crystalline and dislocation free. The images shown in Fig. 2a–c were taken 8 seconds apart. It is seen that upon loading dislocations emerged at GB_1–2_, as indicated by the arrows in Fig. 2b and c. These dislocations are apparently full dislocations. With further straining, dislocations “1”and “4” moved into the interior of G_1_, while dislocations “2” and “3” remained at GB_1–2_. During the process, G_1_ and G_2_ exhibited no obvious fringe changes, indicating that there was no global tilt of the specimen during the mild deformation. Figure 2d provides an enlarged HRTEM image of the framed region shown in Fig. 2b. The extra half atomic plane of dislocation “1” is seen to be along {111}, indicating that it is a 60° mixed full dislocation. Figure 2e is an enlarged HRTEM image of the framed region shown in Fig. 2c, which shows all the four 60° full dislocations more clearly.

**Figure 3:**
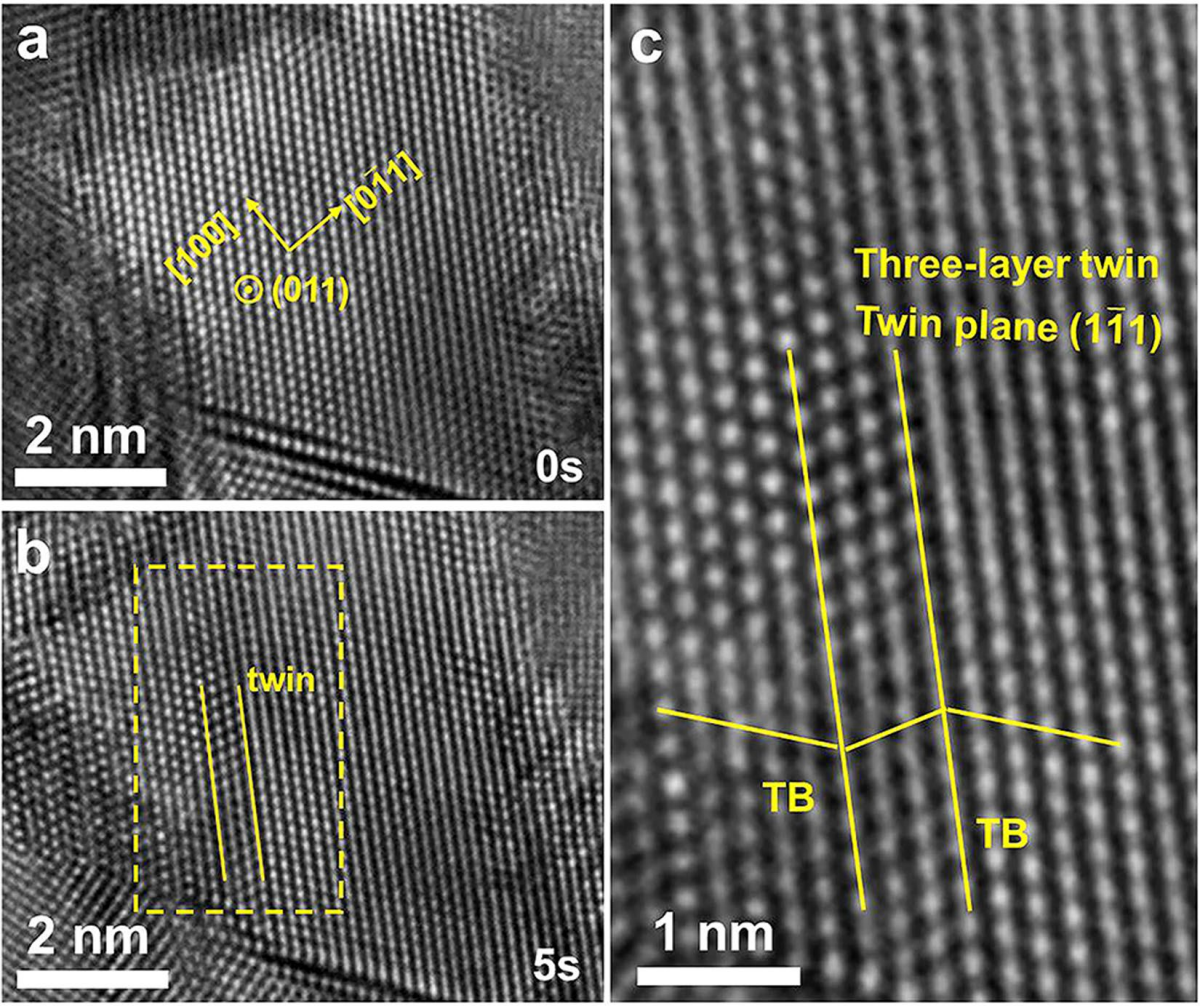
A deformation twin formed in a small grain upon straining in film II, demonstrating the occurrence of partial dislocation formation. The size of the grain is ~8 nm. No full dislocation activity was observed in this grain.

Figure 3 shows the deformation process of film II. As seen in Fig. 3a, the grain with grain size of ~8 nm is dislocation free in the center. Figure 3b is a HRTEM image taken 5 seconds after the start of straining, at which moment a deformation twin was nucleated, implying the creation of partial dislocations. Figure 3c shows the framed region in Fig. 3b at a higher magnification, revealing the deformation twin in more detail.

**Figure 4:**
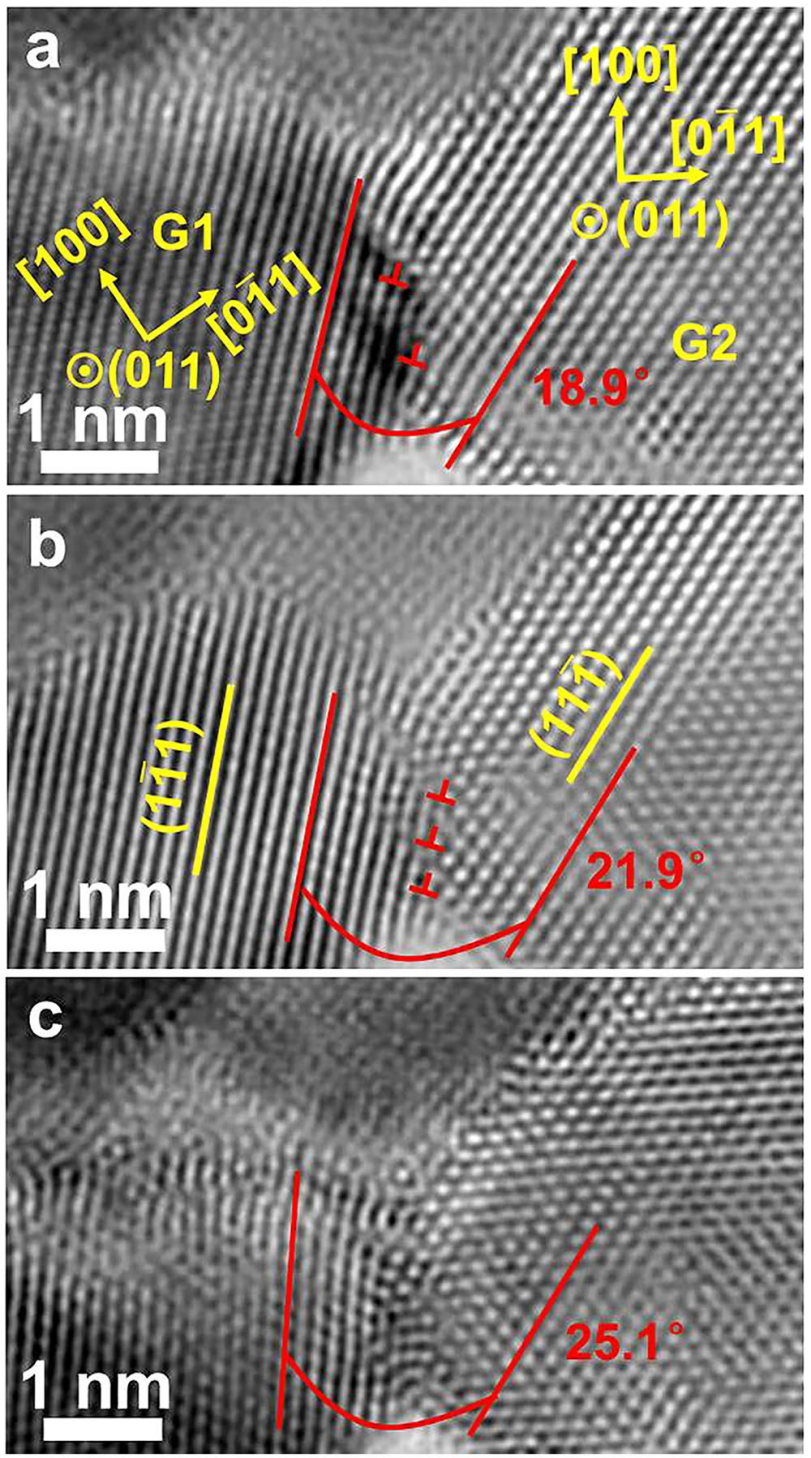
HRTEM images of film II during straining, showing grain boundary dislocation-mediated grain rotation process observed at the atomic scale. Images (**a**), (**b**) and (**c**) were taken with progressive increase of strain.

The evidence presented above demonstrates that full and partial dislocations occur in both films, implying dislocation-mediated plasticity. For film II, in addition to the partial dislocations (deformation twins), grain rotation was also observed among the nano-sized grains. Figure 4 shows a series of HRTEM images revealing grain boundary dislocation-mediated grain rotation in film II. Two grains are seen in Fig. 4a, labelled G_1_ and G_2_. Both grains are found to be below 6 nm in size. The two grains happen to be in [110] axis orientation and share a title grain boundary of 18.9°. During straining, the number of dislocations at G_1–2_ increased and the average spacing of the dislocations on the grain boundary decreased, as seen in Fig. 4b. This leads to an increase of the orientation angle between the two grains from 18.9° to 21.9°. No dislocations were observed inside the grains throughout the deformation process. With further straining, the original dislocation-structured grain boundary transformed into a typical high-angle grain boundary (of 25.1°) with a much less ordered structure, as seen in Fig. 4c. During the straining, G_1_ and G_2_ exhibited no obvious lattice contrast change, indicating that the observed grain rotation is not caused by a global tilt of specimen during deformation. The occurrence of grain rotation implies a deviation from the dislocation-grain boundary interaction controlled plasticity, i.e., the Hall-Petch relationship. Thus it indicates the onset of an inverse Hall-Petch relationship in this nanocrystalline Pt thin film with grain sizes below 6 nm^26,29,32^.

**Figure 5:**
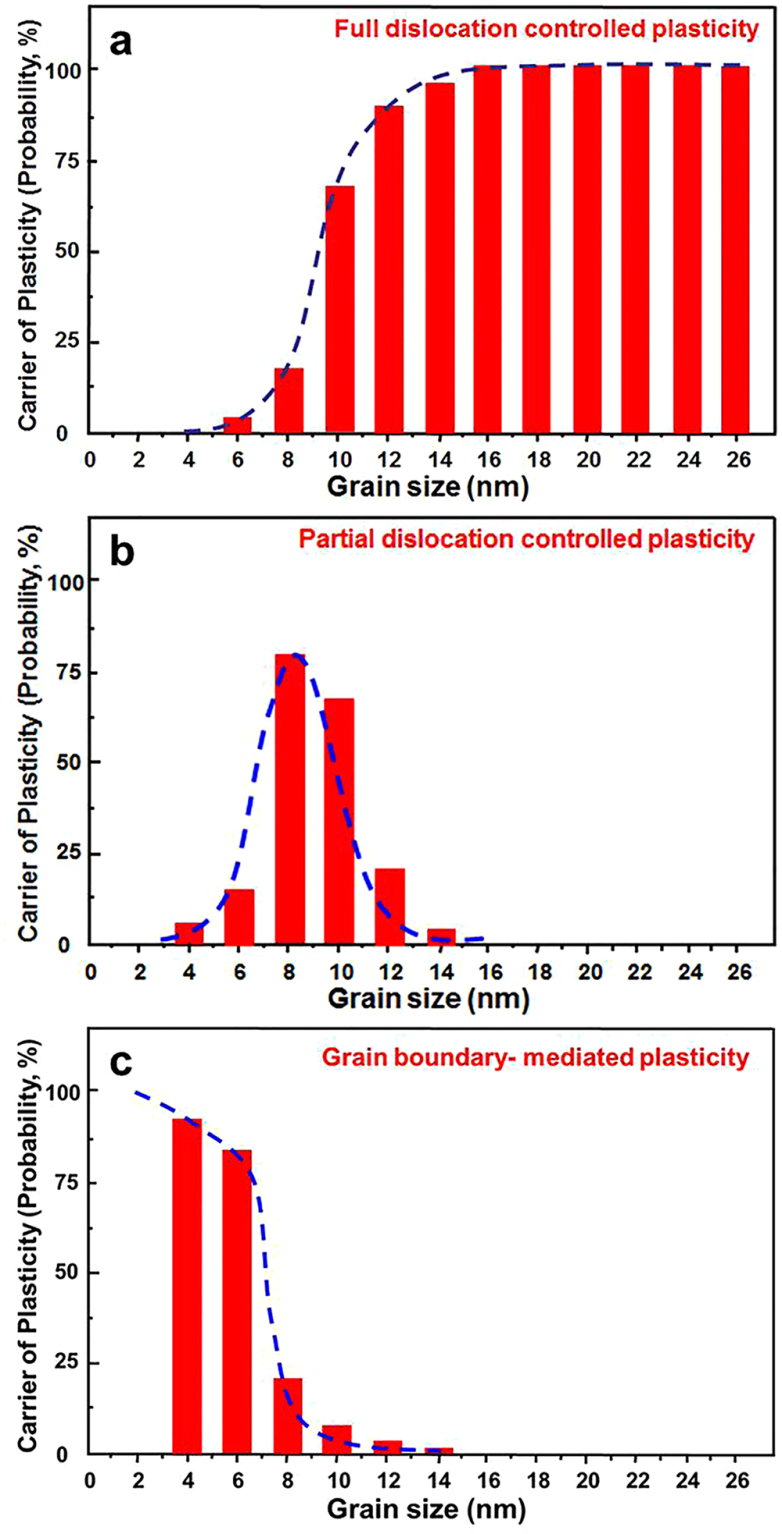
Statistics of grain size-dependence of plastic deformation mechanisms of nanocrystalline Pt thin films. (**a**) Frequency of occurrence of full dislocations. (**b**) Frequency of occurrence of partial dislocations. (**c**) Frequency of occurrence of grain boundary-mediated plasticity.

More than 80 grains (with *d* ranging from 4 to 26 nm) were monitored during the straining process. Figure 5 shows the statistical results of the grain size-dependence of plastic deformation mechanisms of nanocrystalline Pt thin films. It is seen that full dislocation activity is the dominant mechanism of plasticity in nanocrystalline Pt thin films with grains larger than 10 nm (Fig. 5a). In these samples, we frequently observed movements and interactions of cross-grain full dislocations; while partial dislocations and grain rotation are rarely observed. For grains smaller than 6 nm, the dominant mechanism is grain rotation, i.e., grain boundary-mediated plasticity (Fig. 5c). In between for grains of 6 ~ 10 nm in size, partial dislocations were frequently observed (Fig. 5b). These observations present a clear transition of plasticity mechanisms with decreasing grain size from full dislocations, to partial dislocations, and finally to grain rotation.

### Transition from full dislocation to partial dislocation activities for plasticity

It is evident in Fig. 5 that there is a transition from full dislocation activity to partial dislocation activity as the main mechanism of plasticity when the grain size is reduced to a critical value. To understand this grain size dependence of plasticity in nanocrystalline metals, Chen *et al*. calculated the critical grain size for the transition based on the critical shear stress required for the nucleation of these dislocations^1,32^. For a Frank–Read type grain boundary dislocation source^10^, the critical shear stress required for dislocation nucleation can be considered as the required shear stress to induce a strain of the magnitude of a single Burgers vector in a grain of size *D*, i.e.^1,32^:

1 $${\tau }_{N}=\frac{{\rm{2}}\alpha \mu |\mathop{{b}_{N}}\limits^{\longrightarrow}|}{D}$$

2 $${\tau }_{P}=\frac{{\rm{2}}\alpha \mu |\mathop{{b}_{P}}\limits^{\longrightarrow}|}{D}+\frac{\gamma }{|\mathop{{b}_{P}}\limits^{\longrightarrow}|}$$

where ${\tau }_{N}$ and ${\tau }_{P}$ are the critical nucleation shear stresses for a full dislocation and a partial dislocation respectively, $\alpha$ is a parameter reflecting the character of the dislocation ($\alpha$ = 0.5 for an edge dislocation and $\alpha$ = 1.5 for a screw dislocations), $|\mathop{{b}_{N}}\limits^{\longrightarrow}|$ and $|\mathop{{b}_{P}}\limits^{\longrightarrow}|$ are the magnitudes of the Burgers vectors of the full and partial dislocations respectively, $\gamma$ is the stacking fault energy and $\mu$ is the shear modulus. To nucleate a partial dislocation, it also creates a stacking fault, thus the addition of the second term of $\frac{\gamma }{|\vec{{b}_{P}}|}$ in equation (2). It is seen that both stresses are linear functions of 1/D, with ${\tau }_{N}$ having a larger positive slope than ${\tau }_{P}$ because $|\overrightarrow{{b}_{N}}| > |\overrightarrow{{b}_{P}}|$. This is easy to understand intuitively that a partial dislocation imposes a much less lattice elastic distortion energy than does a full dislocation, thus is much easier to be accommodated within a nano-sized grain. It is also apparent that these linear relationships only hold true when D is below a threshold value. Considering the contribution of the grain size independent term $\frac{\gamma }{|\vec{{b}_{P}}|}$, it is easy to see that for coarse grains ${\tau }_{P} > {\tau }_{N}$ and that the two lines cross each other to become ${\tau }_{P} < {\tau }_{N}$ the grain size is reduced to below a critical value *D*_*c*_, which can be determined by equating equations (1) and (2), as expressed below:

3 $${{D}}_{{c}}=\frac{{\rm{2}}\alpha \mu (|\mathop{{b}_{N}}\limits^{\longrightarrow}|-|\mathop{{b}_{P}}\limits^{\longrightarrow}|)|\mathop{{b}_{P}}\limits^{\longrightarrow}|}{\gamma }$$

For Pt, with a stacking fault energy *γ* of 0.27~0.373 J/m^33,34^, a shear modulus *μ* of 65.4 GPa^35^, $|\mathop{{b}_{N}}\limits^{\longrightarrow}|$ = 0.28 nm, $|\mathop{{b}_{P}}\limits^{\longrightarrow}|$ = 0.16 nm, and taking $\alpha$ = 1, the critical grain size *D*_*c*_ is estimated to be 6.7~9.3 nm. This value is consistent with our experimental observation that full dislocation activities prevail as the dominant plastic deformation mechanism of nanocrystalline Pt thin film only with grain sizes above 10 nm.

### Grain boundary-mediated plasticity

It is also evident above that when the grain size is further reduced to 6 nm and below, dislocation activities are suppressed. In the absence of dislocation activities, other mechanisms have to operate to provide the required plastic deformation. These may include grain creep, grain sliding and grain rotation. All of the three mechanisms are thermally activated processes and involve self-diffusion of atoms under the influence of a bias stress^36–39^.

#### Stress-assisted grain creep

Grain creep is a grain deformation process caused by stress-assisted atomic diffusional migration. The atomic migration may occur via three routes, i.e., migration through the lattice, referred to as the Nabarro-Herring creep, migration along grain boundaries, referred to as the Coble creep, and migration through surface diffusion. Their contributions to plastic strain rate may be expressed as:

4 $${e}_{gc}=\frac{{\rm{14}}W\sigma {(D}_{{\rm{l}}}+{{D}}_{{s}})}{kT{d}^{{\rm{2}}}}+\frac{\mathrm{14}{\rm{\pi }}W\sigma \delta {D}_{gb}}{kT{d}^{{\rm{3}}}}$$

where *W* is the atomic volume (10^−29^ m^3^), *σ* is the stress, *d* is the grain size, *k* is Boltzmann constant, *T* is the testing temperature, *D*_*l*_ is the coefficient of self-diffusion through the lattice, *D*_*s*_ is the coefficient of self-diffusion along the surface, *D*_*gb*_ is the coefficient of self-diffusion along the grain boundary, and *δ* is the grain boundary width. The first term expresses the contribution of Nabarro-Herring creep *e*_*N*_ and surface diffusion *e*_*s*_ and the second term expresses the contribution of Coble creep *e*_*c*_. Although both of free surface and grain boundary are interfaces, atoms on surface mainly exist within grains. Therefore, the surface diffusion could be taken as a process within grains rather than at grain boundaries, which means that grain boundary width could be neglected as in first term of equation (4). Self-diffusion coefficient should be the only variant of plastic strain rate once the stress, grain size and temperature determined for the same material. Therefore, the contribution of surface diffusion to plastic strain rate could be estimated by adding *D*_*s*_ to the function of Nabarro-Herring creep. It should be noted that the constant of the first term in equation (4) is empirical and thereby could be different for Nabarro-Herring creep and migration through surface diffusion. However, the change of constant can hardly result in the variation of several magnitudes of strain rate, which means that the result can be still reasonable for a rough estimation of strain rate.

*D*_*l*_, *D*_*s*_ and *D*_*gb*_ can be computed using *D* = *D*_*o*_ *exp*(*−Q/RT*) for coarse-grained metals^40^. For *D*_*l*_, *D*_*o*_ is 5.4 × 10^−5^ m^2^/s for FCC metals and *Q* is 153*T*_*m*_ for lattice diffusion^40^. For this study the *in situ* deformation temperature inside the TEM is estimated to be 353 K. With these parameters, *D*_*l*_ is estimated to be 5.24 × 10^−52^ m^2^/s. For *D*_*s*_, *D*_*o*_ is 2.2 × 10^−5^ m^2^/s for FCC metals and *Q* is 62.7*T*_*m*_^37^. Therefore, *D*_*s*_ is estimated as 1.2 × 10^−24^ m^2^/s. For *D*_*gb*_, *D*_*o*_ is 9.7 × 10^−6^ m^2^/s for FCC metals and *Q* is 75.4*T*_*m*_ for grain boundary diffusion^40^. *D*_*gb*_ is thus estimated to be 6.58 × 10^−29^ m^2^/s. It has been suggested that *D*_*gb*_ for nanocrystalline metals can be 3 orders of magnitude larger than that for coarse-grained counterparts due to the difference in the grain boundary density^41–44^. Thus, *D*_*gb*_ for the nanocrystalline Pt thin films is assumed to be 6.58 × 10^−26^ m^2^/s. The stress applied to the sample is not measured in the tensometer. Referring to examples in the literature, a yield strength of *σ* ≈ 1 GPa)^29,45^ for nanocrystalline metals is adopted. Using this stress, *d* = 6 nm and *δ* = 0.5 nm, the computed strain rate contribution of Coble creep is *e*_*C*_ = 1.3 × 10^−8^/s for the nanocrystalline Pt thin films. The computed strain rate contribution of Nabarro-Herring creep is *e*_*N*_ = 4.2 × 10^−34^/s, which is negligible to the total grain creep *e*_*gc*_. The estimated strain rate contribution of surface diffusion is 9.6 × 10^−7^/s. This strain rate contribution is more than one order of magnitude higher than that from Coble creep. This means that surface diffusion is the dominant mechanism of grain creep. The total grain creep strain rate is *e*_*gc*_ ≈ *e*_*c*_ + *e*_*s*_ = 9.73 × 10^−7^/s.

It is apparent that this value is far smaller than the experimental strain rate of 1 × 10^−3^/s. This implies that grain creep may not be the dominant mechanism of the observed plastic deformation of the nanocrystalline Pt thin film at *d* < 6 nm.

#### Grain rotation

**Figure 6:**
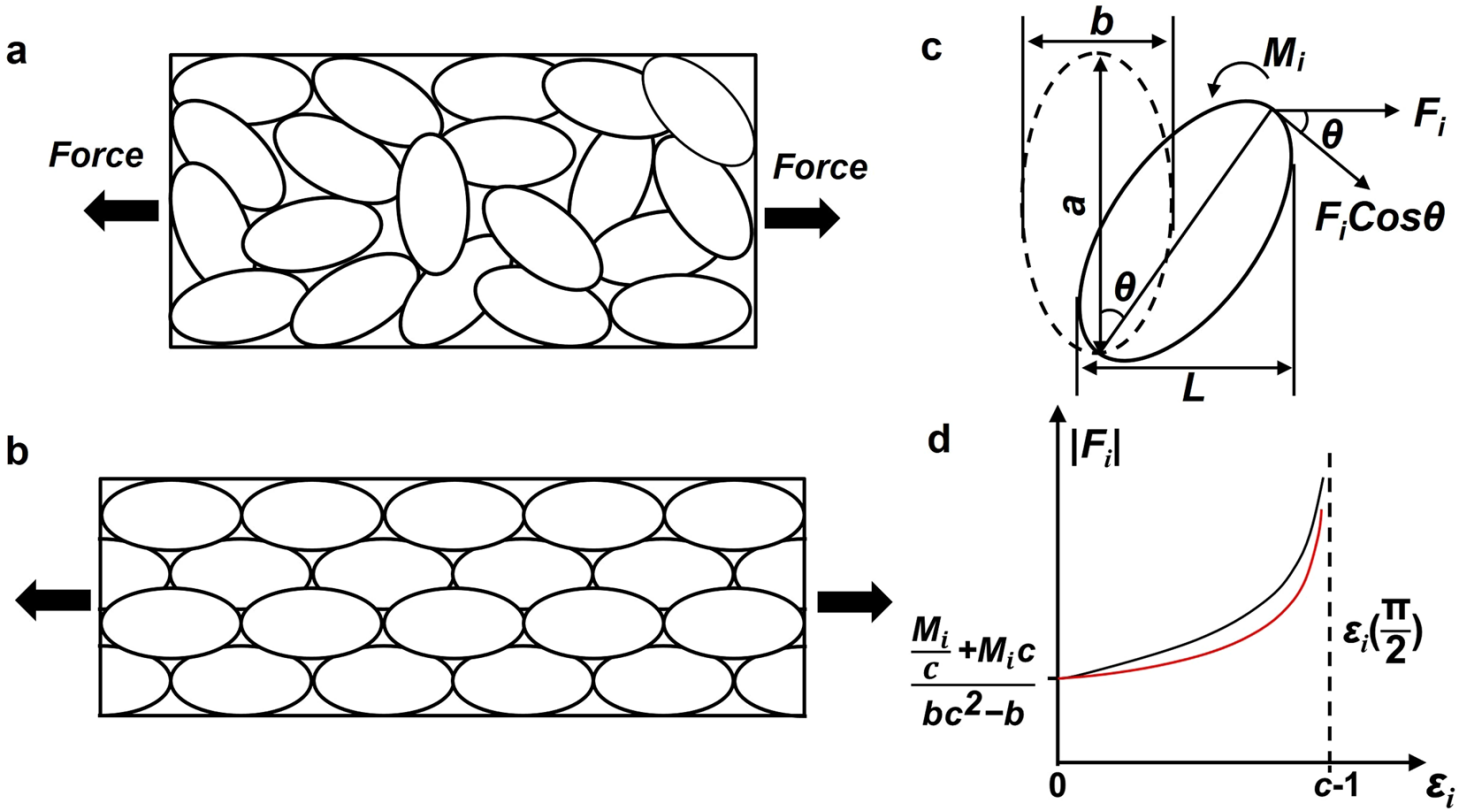
Schematics of the plastic strain induced by grain rotation under tensile stress. To show the aspect ratio of grains more vividly, the grains are oval-shaped. (**a**) Original distribution of nanograins. (**b**) Distribution of nanograins after plastic deformation under force *F*. (**c**) A spheroid grain with axes *a* and *b* rotating under the influence of force *F*_*i*_. (**d**) The |*F*_*i*_|–*ε*_*i*_ curve under the effect of a constant critical moment resistance *M*_*i*_.

Stress-induced grain rotation is another mechanism of deformation^44^. Grain rotation may occur by the mechanisms of grain boundary diffusion^44^ and activities of grain boundary dislocations^16^. Figure 6 shows schematics of grain rotation induced by a tensile stress. Under this condition the oval-shaped grains rotate to align their longer axes towards the direction of the tensile stress (or away from it if it is a compressive stress), so to produce the maximum strain (as shown in Fig. 6a–b). The strain contribution of the rotation of a grain can be computed based on the geometrical parameters of the grain, including the aspect ratio (*c* ≠ 1) and the initial angular position (*θ*). To further estimate the force required for the rotation, a critical and constant moment resistance (*M*_*i*_) as exerted by grain boundaries can be assumed.

Figure 6c–d shows a schematic of grain rotation for force-strain analysis. *F*_*i*_ is the external force on the *i*th grain. The grain in analysis is a spheroid with a longer axis *a* and a shorter axis *b* (and *c* = *b*/*a*), and at an initial position of *θ* = 0. The critical force required for rotation of an individual grain may be expressed as:

5 $${F}_{i}={M}_{i}/(aCos\theta )$$

The plastic strain *ε*_*i*_ of the *i*th grain, or the relative elongation the rotation produces in the direction of the force, can be expressed as:

6 $${\varepsilon }_{i}=\frac{aSin\theta +bCos\theta }{b}-{\rm{1}}$$

It is seen that both *F*_*i*_ and *ε*_*i*_ are functions of *θ* and that *ε*_*i*_ reaches its maximum at *θ* = π/2. Solving the equations to eliminate *θ* leads to the following relationship between *F*_*i*_ and *ε*_*i*_:

7 $$\frac{\frac{{M}_{i}}{c}-{M}_{i}}{\frac{b{c}^{{\rm{2}}}}{{\varepsilon }_{i}+1}-b({\varepsilon }_{i}+1)}+\frac{{M}_{i}(c+1)}{b{c}^{{\rm{2}}}-b({\varepsilon }_{i}{+1)}^{{\rm{2}}}} < |{F}_{i}| < \frac{{{\rm{M}}}_{{\rm{i}}}+\frac{{M}_{i}}{c}}{\frac{b{c}^{{\rm{2}}}}{{\varepsilon }_{i}+1}-b({\varepsilon }_{i}+1)}+\frac{{M}_{i}(c-1)}{b{c}^{{\rm{2}}}-b({\varepsilon }_{i}{+1)}^{{\rm{2}}}}$$

This relationship expresses the lower and upper bound conditions for the applied force, as described by the left and right inequations respectively, which are defined by mathematical approximations of a complex square root term in the calculation. Figure 6d plots this relationship. It is seen that |*F*_*i*_| increases rapidly to infinity when *θ* → π/2 and that *ε* has a maximum limit of *c*−1 at *θ* = π/2. This explicitly demonstrates that grain rotation may be a major contribution to plastic deformation only at the very early stage of deformation but cannot be the main mechanism of plastic deformation at high strain levels.

Figure 6d (and inequation (7)) implies that grain rotation cannot occur when the external force is below the lower bound curve. This is based on the assumption that the critical resistance to rotation (*M*_*i*_) is a constant finite value, which describes a static condition. For a grain boundary-mediated deformation process, stress-assisted atomic migration via diffusion along grain boundaries provides the mechanism of deformation^44^. Stress-assisted atomic diffusion is a kinetic process and requires no minimum stress. Instead, a power law can be suggested to describe relationship between the rotation angular velocity *ω* and the moment *M:*

8 $$\omega =\frac{\theta }{t}=k{M}^{n}=k{(\frac{{F}_{i}}{aCos\theta })}^{n}$$

where *t* is the rotation time, *k* is the coefficient parameter and *n* is a power parameter. It is reasonable to suggest that parameter *k* is largely dependent on the grain boundary diffusion coefficient *D*_*gb*_. This equation expresses that there is no minimum applied load for diffusion-enabled grain rotation and that the angular velocity of grain rotation increases with increasing the applied force.

Summarizing the considerations of equations (5), (6) and (8), the following can be concluded:

1. Grain rotation may occur under an applied uniaxial stress only when *c* ≠ 1.
2. There exists a maximum plastic strain that grain rotation can generate, limited to *ε*_*i*_ = *c*−1, or *θ* = π/2 for rotation angle.
3. The applied stress required for grain rotation increases rapidly during the process of grain rotation, or equivalently to say, the strain rate due to grain rotation under a constant applied load decreases rapidly during the process of grain rotation.
4. The strain rate contribution from grain rotation is dependent on several factors, including the mean grain aspect ratio, the mean initial angular position of the grains, the applied load, and the diffusion coefficient (and temperature).

It also needs to be pointed out that the Pt thin films used in this study contained equiaxial nanograins. Thus grain rotation cannot be the dominant mechanisms of plastic deformation of the Pt thin films in this work. However, grain rotation was observed *in situ* during deformation inside the TEM in this study. This is attributed to the need to accommodate plastic deformation by grain sliding, as explained below, instead of being a direct contribution to the deformation strain. In other words, grain rotation in this work is not the main plastic deformation mechanism to provide plastic strain.

#### Grain sliding

Nanograins may slide one from another along their boundaries to align to the direction of a tensile load, thus to produce a strain. The strain rate associated with this process is controlled by the rate of stress-assisted atomic migration via diffusion along the grain boundaries, which can be expressed as^46^:

9 $${e}_{gs}=\frac{{2\times \mathrm{10}}^{{\rm{5}}}{D}_{gb}\mu |\vec{b}|}{kT}{(\frac{|\vec{b}|}{d})}^{{\rm{3}}}{(\frac{\sigma }{\mu })}^{{\rm{2}}}$$

where $|\vec{b}|$ is the magnitude of the Burgers vector of grain boundary dislocations. Using the relevant parameters presented above, ${e}_{{gs}}$ is estimated to be 1.13 × 10^−6^/s. This strain rate is comparable in magnitude to the strain rate contribution of grain creep (9.73 × 10^−7^/s). This implies that both grain sliding and surface diffusion-dominated grain creep may serve as the main contributing mechanisms of plastic deformation of the nanograined thin film. However, these plastic strain rate contributions are still three orders of magnitude lower than the experimentally observed strain rate.

A possible explanation of such discrepancy may reside in the value of *D*_*s*_ and ${D}_{{gb}}$ used. Most studies^38–42,46^ on strain rate contribution of grain sliding mainly consider the thermal factor of the diffusion activation energy *Q* in the Ahrrenius equation *D* = *D*_*o*_ *exp(−Q/RT)*. Factor *σ* in equations (4) and (9) only expresses the impact of stress on mechanical work but neglects the influence of stress on diffusional coefficient *D*_*l*_, *D*_*gb*_ and *D*_*s*_. It is almost fundamentally obvious to expect that an applied stress has direct influence in promoting atomic migration via diffusion, which can be reflected as its effect on the diffusion activation energy^47^. To take this effect into account, the Ahrrenius equation for diffusion coefficient under the influence of an applied stess may be expressed as:

10 $$D={D}_{o}exp(-(Q-{Q}_{s})/RT)$$

where *Q*_*s*_ is the activation energy term caused by the applied stress.

However, *D*_*l*_ is too small to have significant influence on the plastic strain rate compared with *D*_*gb*_ and *D*_*s*_, even when the effect of stress is taken into account. The effect of stress should be considered for *D*_*gb*_ and *D*_*s*_, which have comparable contributions to plastic strain rate. As pointed out before, a small underestimation of the diffusion activation energy, e.g., by neglecting the influence of stress, can lead to possible large discrepancy between the theoretical prediction and the experimentally measured strain rate. The role of stress in mechanical work is already considered in equations (4) and (9), which also determine the main direction of plastic deformation through *D*_*s*_-dependent surface diffusion and *D*_*gb*_-dependent grain sliding. The contribution of external stress to activation energy for diffusion, which determines *D*_*gb*_ and *D*_*s*_, mainly stemps from its effect on enhacing the chemical potential of atoms^48^, akin to the effect of an increased temperature. A stressed atom is more energized and less stable, thus easier to break its bonds to migrate via diffusion. This is equivalent to a decrease in the activation energy for diffusion, i.e., Q-Q_s_. Using the experimentally applied strain rate of 1 × 10^−3^/s, ${D}_{{gb}}$ can be estimated using equation (9) to be 5.6 × 10^−23^ m^2^/s. This corresponds to a *Q*_*s*_ of 1.98 × 10^4^ J/mol according to equation (10) (here, *D*_*o*_ for *D*_*gb*_ is adopted as 9.7 × 10^−3^ m^2^/s). This value is approximately one order of magnitude lower than *Q*, which is 1.6 × 10^5^ J/mol. *Q*_*s*_ is clearly a function of stress, but the exact relationship is yet to be established. To apply *Q*_*s*_ into the first term in equation (4), the contribution of surface diffusion to strain rate (as *D*_*l*_ is negligible compared with *D*_*s*_) is computed as 8.08 × 10^−4^ /s, which is comparable with that of grain sliding. It implies that the contribution of surface diffusion to strain rate is as significant as that of grain sliding.

In light of the modified ${D}_{{gb}}$ under the influence of an applied stress, the strain rate contribution of grain creep discussed abive ought also to be re-examined to assess its validity as a main deformation mechanism. Using ${D}_{{gb}}$ = 5.6 × 10^−23^ m^2^/s in equation (4), the strain rate contribution due to grain creep under the influence of stress is estimated to be 6.2 × 10^−5^/s, which is still two orders of magnitude smaller than experimental strain rate.

Summarizing the above analyses, it is seen that both of grain creep and grain sliding can serve as the main mechanisms of plasticity for nanocrystalline Pt thin films. In comparison, grain rotation cannot be a main mechanism of plasticity due to its geometrical limitation to strain, particularly for matrices of equiaxial grains.

## Conclusions

The experimental evidence and the analyses presented above enable the following conclusions to be reached:

1. This study revealed direct evidence of grain size dependence of plasticity mechanisms in nanocrystalline Pt thin films. The dominant mechanism of plasticity was found to change from full dislocation activity at grain sizes of *d* > 10 nm, to partial dislocation activity at grain sizes of 10 nm > *d* > 6 nm, and grain boundary-mediated deformation at grain sizes of *d* < 6 nm.
2. At the absence of dislocation activities, other diffusion-enabled deformation mechanisms were examined. Analysis using established strain rate models indicates that grain creep via diffusion can offer considerable strain rate by surface diffusion of atoms.
3. An analysis was made on the possibility of grain rotation as a mechanism of plasticity. The analysis demonstrates that the contribution of grain rotation is dependent on several factors, including the mean grain aspect ratio, the mean initial angular position of the grains, the applied load, and the diffusion coefficient (and temperature). The strain contribution of grain rotation is only possible with nanograins of aspect ratio *c* ≠ 1 (i.e. non-equiaxial grains). It also demonstrates that there is maximum strain that grain rotation may produce, limited to *c* − 1 and that the strain rate due to grain rotation under a constant applied load decreases rapidly during the process of grain rotation.
4. Grain rotation was observed *in situ* during tensile deformation of the equiaxial nanograin Pt thin films in this study. This is attributed the need to accommodate plastic deformation via grain sliding.
5. Grain sliding can provide comparable strain rate with grain creep. Both are considered as plausible dominant mechanisms for plastic deformation of nanocrystalline Pt thin films. Grain sliding may be accompanied by rotation of grains due to the frictional sliding along the grain boundaries.

## Methods

**Figure 7:**
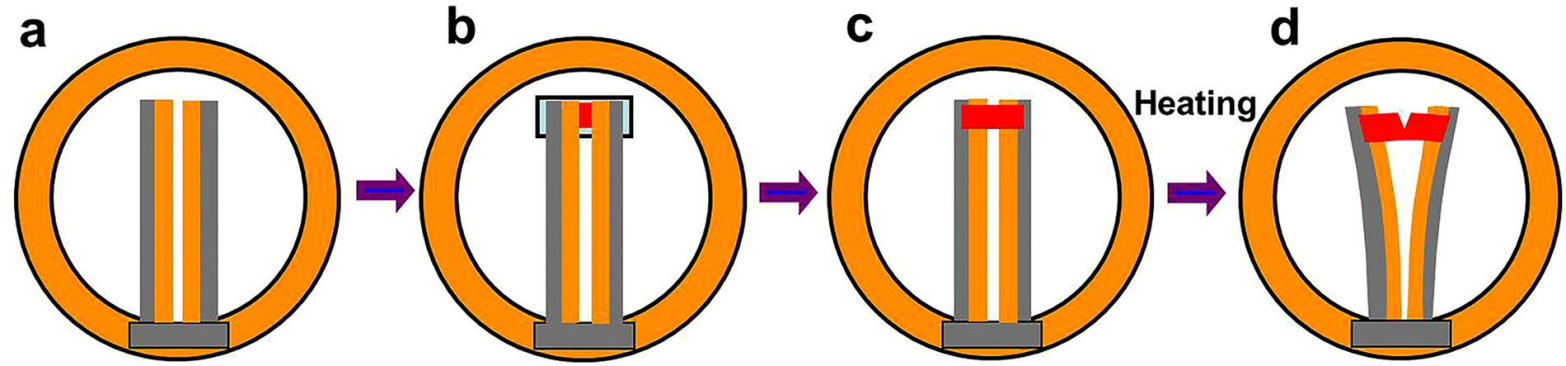
Schematic of the *in situ* tensile testing device. The dimensions of the parts are not in proportion. (**a**) The home-made bi-metal tensometer mounted on a ϕ3 mm TEM Cu ring. (**b**) A small rectangular piece of thin film-substrate sample is attached with epoxy on to the end of the bi-metal arms of the tensometer with the film side in contact with the arms (bottom view). (**c**) Upon dissolving away the NaCl substrate in pure water, the free-standing thin film is released and left attached to the arms of the tensometer. (**d**) Tensile straining of the film sample induced by heating the bi-metal arms of the tensometer in a conventional TEM hot stage sample holder.

A Pt nanocrystalline thin film of ~10 nm in thickness was deposited by means of magnetron sputtering on a (001) surface-oriented NaCl single crystal substrate (30 × 30 mm^2^) at 300 °C. Specimens for *in situ* deformation TEM analysis were in a small rectangular shape of 4 × 2 mm in dimension, cut off from the film-substrate sample using a sharp blade. The specimen was attached to a home-made bi-metal tensometer for *in situ* tensile deformation, as schematically shown in Fig. 7. The specimen was attached to the tensometer using epoxy resin, with the Pt thin film side facing down in contact with the bi-metal arms of the tensometer, as schematically shown in Fig. 7b. The NaCl substrate was then removed by dissolving into pure water to liberate the Pt thin film sample on the bi-metal arms of the tensometer, as show in Fig. 7c. The tensometer with the thin film sample was then mounted on to a conventional TEM double tilt hot stage sample holder. The bi-metal arms of the tensometer can be activated to bend outward upon joule heating to generate a tensile displacement on the thin film sample (Fig. 7d). The gauge section of the film sample is 0.2 × 2 mm with tension in the width direction (~0.2 mm).

The *in situ* tension was applied to the Pt thin film by slowly heating up the bi-metal arms of the tensometer using a Gatan heating holder inside the microscope. The temperature required for deformation was up to 80 °C and strain rate was dependent on the heating rate and was estimated to be approximately 10^–4^/s^27–30^. This temperature range (25 °C–80 °C) is expected to have negligible impact on the deformation mechanisms that may operate in the sample, considering the high melting point of Pt (1768 °C). The real-time atomic movement during deformation was examined using a JEM-2010F TEM, operated at 200 kV.
